# Supplementary material for: Attentional fluctuations induce shared variability in macaque primary visual cortex
Source: Nat Commun. 2018 Jul 9;9:2654. doi: 10.1038/s41467-018-05123-6 (PMC6037755; doi:10.1038/s41467-018-05123-6)
Supplement: Supplementary file 1 — Supplementary Information [file 41467_2018_5123_MOESM1_ESM.pdf]

Attentional fluctuations induce shared variability in macaque primary visual cortex

Denfield et al.

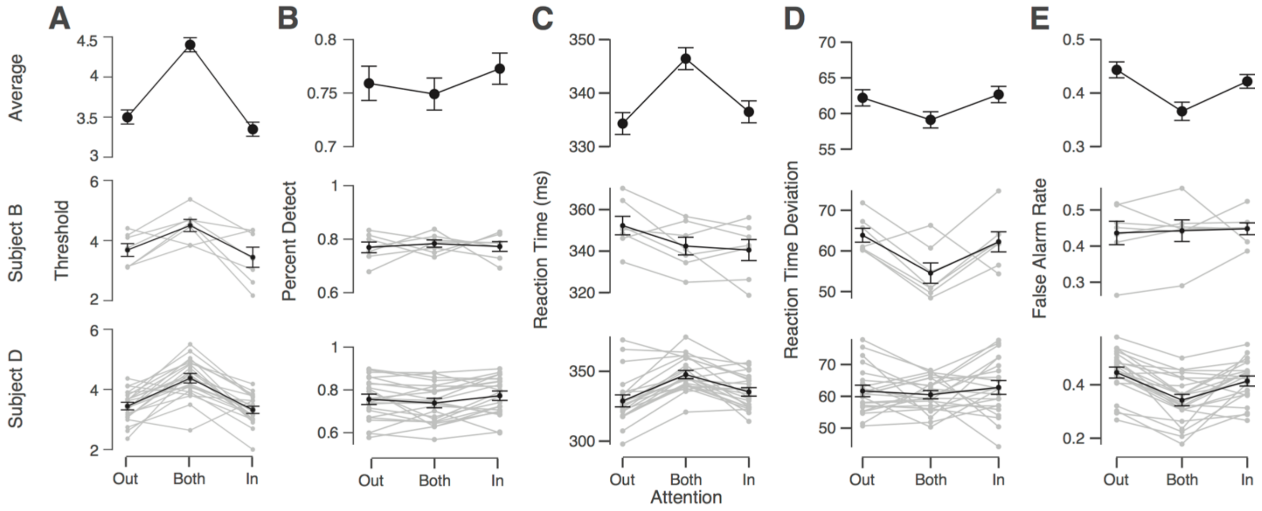

Supplementary Figure 1. Behavioral results for each subject and session.

Black lines show mean across sessions with error bars representing SEM. Lighter gray lines show individual session results. **A)** 50% detection thresholds averaged across all sessions (top), for Subject B sessions only (middle), and for Subject D sessions only (bottom). **B)-E)** show percent detect, reaction times, reaction time median deviations, and false alarm rates, respectively, using a similar organization as panel **A**.
